# Comparison of Naringenin and Alendronate in the Attenuation of Ovariectomy-Induced Osteoporosis by Modulating Autophagy, Apoptosis, and Oxidative Stress in Rats

**DOI:** 10.5812/ijpr-171182

**Published:** 2026-06-13

**Authors:** Hongwei Gao, Pengcheng Ma, Huizhi Chen, Zhengkai Zhang, Nara Davtyan, Jiachun Zheng

**Affiliations:** 1 Department of Orthopaedics, Public Health Clinical Center Affiliated to Shandong University, Jinan, China; 2 Yerevan State University, Yerevan, Armenia

**Keywords:** Osteoporosis, Ovariectomy, Naringenin, Alendronate, Autophagy, Apoptosis, Oxidative Stress, Stereology

## Abstract

**Background:**

Estrogen deficiency after menopause is a major driver of osteoporosis and has been increasingly linked to dysregulated cellular stress responses, including autophagy and apoptosis, that affect bone remodeling and microarchitecture.

**Objectives:**

This study aimed to evaluate the therapeutic effects of naringenin (NA) and alendronate (AL) on ovariectomy (OVX)-induced osteoporosis in rats, with emphasis on autophagy- and apoptosis-related molecular signatures.

**Methods:**

Female Sprague-Dawley rats were assigned to sham-operated (SH), OVX, OVX+NA (50 mg/kg/day), or OVX+AL (5 µg/kg/day) groups (n = 10/group) and were treated by oral gavage for 10 weeks. Bone health markers, including bone cell numbers and trabecular weight and volume, were assessed using stereology. Serum estradiol and osteocalcin levels were assayed by enzyme-linked immunosorbent assay. Oxidative markers, including catalase (CAT) and glutathione reductase (GR) activities, were measured using spectrophotometric assays. Femoral mRNA expression of microtubule-associated protein 1 light chain 3 (LC3), beclin 1 (BECN1), autophagy-related 5 (ATG5), caspase 9 (CASP9), and B-cell lymphoma 2 (BCL2) was quantified by reverse transcription quantitative polymerase chain reaction.

**Results:**

Ovariectomy reduced estradiol levels compared with SH, and estradiol was not restored by NA or AL. Both treatments mitigated OVX-associated changes in osteocalcin and improved femoral stereological/histomorphometric outcomes, with AL exerting a greater effect across multiple structural and molecular outcomes. NA, but not AL, significantly increased CAT and GR activities relative to OVX. Ovariectomy increased CASP9 and autophagy markers and decreased BCL2; both treatments shifted these transcripts toward SH values, with a stronger effect for AL.

**Conclusions:**

Naringenin and AL mitigated OVX-associated osteoporotic changes without restoring systemic estradiol levels, and these effects were accompanied by shifts in apoptosis- and autophagy-related gene expression. These findings suggest that the beneficial effects of AL and NA may be associated with the modulation of oxidative stress and apoptosis- and autophagy-related mechanisms.

## 1. Background

Osteoporosis is a prevalent metabolic bone disorder characterized by reduced bone mass and microarchitectural deterioration, culminating in increased skeletal fragility and fracture risk (1-3). Among its major forms, postmenopausal osteoporosis is closely linked to estrogen deficiency, which shifts bone remodeling toward net bone loss through coordinated changes in osteoclast-mediated resorption, osteoblast-driven formation, and osteocyte signaling within the bone multicellular unit (2-6). Given the substantial lifetime burden of osteoporotic fractures and their downstream morbidity, prevention and long-term disease control remain central priorities in musculoskeletal medicine and pharmacology (4-7).

Mechanistically, estrogen withdrawal influences bone remodeling through several convergent pathways. Reduced estrogen signaling increases osteoclastogenesis and osteoclast activity through altered cytokine milieus and changes in the receptor activator of nuclear factor-kappa B ligand (RANKL)/osteoprotegerin (OPG) balance, while simultaneously impairing osteoblast function and survival (2, 3, 8, 9). Oxidative stress and inflammatory signaling are increasingly recognized as amplifiers of this remodeling imbalance, particularly in estrogen-deficient states, contributing to the deterioration of trabecular architecture and reduced biomechanical competence (8, 9). In parallel, osteocytes, long-lived mechanosensory cells embedded within the mineralized matrix, coordinate remodeling through paracrine signals that integrate mechanical cues and systemic hormonal inputs; their dysfunction or loss can propagate maladaptive remodeling responses in osteoporosis (2, 8, 9).

Beyond classical remodeling mediators, regulated cell survival programs have received increasing attention as proximate determinants of bone cell fate under estrogen deficiency. Apoptosis, a programmed cell death pathway, affects both the abundance and functional capacity of osteoblast-lineage cells and osteocytes, thereby shaping bone formation and the maintenance of bone quality (2, 8-10). In experimental estrogen deficiency, increased pro-apoptotic signaling has been associated with osteocyte and osteoblast loss, which can further impair mechanotransduction and coupling between resorption and formation (8, 9). Key molecular readouts commonly used to index apoptotic tone include executioner caspase activity, such as caspase 3, and counter-regulatory pro-survival factors, such as BCL2, which together reflect the balance between cell death and survival pressures in tissue microenvironments (9, 11).

Autophagy, a conserved lysosomal degradation pathway essential for organelle quality control and cellular stress adaptation, has emerged as another critical regulator of bone homeostasis (12-15). Basal autophagy supports osteoblast differentiation and mineralization, contributes to osteocyte longevity, and modulates osteoclast function, whereas dysregulated autophagy has been implicated in osteoporosis pathophysiology (12-14). Canonical autophagy machinery components, such as LC3, BECN1, and ATG5, are frequently assessed as molecular proxies for autophagic activity or capacity in bone tissue studies (12-14). Importantly, autophagy and apoptosis are not independent endpoints; they interact through shared upstream stress signals and molecular crosstalk, and shifts in this interplay may determine whether bone cells adapt to estrogen-deficient stressors or undergo irreversible loss (13, 14, 16). Preclinical evidence in ovariectomy-associated bone loss suggests that interventions capable of restoring autophagic homeostasis may, in some contexts, attenuate pro-apoptotic signaling and preserve osteocyte viability, thereby mitigating structural decline (17).

Current clinical strategies for postmenopausal osteoporosis emphasize antiresorptive therapy, with bisphosphonates remaining among the most widely used first-line agents in many treatment algorithms (4-6). Alendronate, a nitrogen-containing bisphosphonate, reduces fracture risk largely by suppressing osteoclast-mediated bone resorption, translating into net gains in bone mineral density and improved structural integrity over time (4-6, 18). However, long-term therapy requires careful risk-benefit calibration, particularly in relation to rare but clinically consequential adverse outcomes such as medication-related osteonecrosis of the jaw and atypical femoral fractures, as highlighted in contemporary syntheses and guideline discussions (18-20). These considerations motivate ongoing interest in complementary strategies that may support bone cell resilience and bone quality, particularly approaches that engage cellular stress-response pathways beyond osteoclast suppression alone (2, 12-14, 18).

Diet-derived bioactives, including flavonoids, have been investigated as candidate adjuncts for skeletal health because of their pleiotropic anti-inflammatory, antioxidant, and cell-signaling effects (21-23). Naringenin, a citrus-derived flavanone, has been reported to influence osteoblastogenesis and osteoclastogenesis in experimental settings, with some studies suggesting anabolic or antiresorptive potential across different models of pathological bone loss (24-27). In ovariectomy-related contexts, NA administration has been associated with the preservation of bone histomorphometric features and improvements in bone-related outcomes without clear uterotrophic effects in certain preclinical reports, supporting its plausibility as a candidate for postmenopausal osteoporosis-relevant investigation (28, 29). Nonetheless, the mechanistic basis for the putative osteoprotective action of NA remains incompletely resolved, particularly regarding how it may modulate the balance between autophagic adaptation and apoptotic loss within bone tissue under estrogen deficiency (12-14, 16, 21, 24, 25).

## 2. Objectives

Against this backdrop, a focused comparative evaluation of NA relative to an established antiresorptive therapy may help clarify whether modulation of autophagy- and apoptosis-linked programs is associated with improvements in structural and biochemical osteoporosis phenotypes. In the ovariectomized rat model, which is widely used to emulate key features of estrogen deficiency-associated bone loss (29-31), integrating serum biochemical indices of bone turnover with femoral histomorphometric readouts and molecular profiling of autophagy/apoptosis markers can provide a cohesive view of tissue-level changes and their underlying regulatory signatures (12-14, 16, 32). Therefore, the present study aimed to evaluate the therapeutic effects of NA and AL on OVX-induced osteoporosis, with an emphasis on their association with autophagy- and apoptosis-related gene expression in bone. We hypothesized that NA and AL would mitigate OVX-induced osteoporotic changes and that these phenotypic effects would be accompanied by shifts in autophagy- and apoptosis-linked molecular signatures consistent with improved bone cellular homeostasis.

## 3. Methods

### 3.1. Animals and Experimental Design

Female Sprague-Dawley rats aged 3 months with regular estrous cycles were used. The inclusion criteria were 3-month-old female rats with regular estrous cycles and no apparent musculoskeletal abnormalities. The exclusion criteria were infection, surgical complications, partial ovariectomy, and any other abnormalities that developed during therapy. None of the rats met the exclusion criteria.

Animals were allocated to 4 groups (n = 10/group)—sham-operated (SH), OVX, OVX+NA, and OVX+AL—using a computer-generated randomization schedule. An investigator not involved in outcome assessment performed the group allocation. Naringenin (≥98% purity) and sodium AL were obtained from Sigma-Aldrich (St. Louis, MO, USA). For oral gavage at 50 mg/kg/day, a fresh NA suspension was prepared in 0.5% carboxymethylcellulose sodium solution. Sodium AL was freshly prepared in distilled water. After surgery, NA was administered at 50 mg/kg/day and AL at 5 µg/kg/day by oral gavage for 10 weeks. A separate investigator not involved in endpoint analyses administered the oral gavage treatments.

To minimize potential cage effects, rats from different treatment groups were distributed across several cages, with the same number of rats per cage and identical environmental conditions, including temperature, humidity, and a 12-hour light/dark cycle. Both compounds were obtained from commercial suppliers and prepared according to the manufacturers’ recommendations.

The sample size was established based on previous studies using OVX rat models of postmenopausal osteoporosis with similar stereological and biochemical endpoints, indicating that 6 to 8 animals per group were adequate to identify significant variations in trabecular bone volume and bone turnover markers (33). The primary outcome measures were bone weight, trabecular bone volume, and trabecular volume density, assessed by stereology, as these parameters were considered the primary determinants related to bone loss and therapy. Secondary outcome measures included blood biomarkers, including osteocalcin, estrogen, calcium, CAT, and GR; bone cell counts; and mRNA expression of genes encoding autophagic proteins (LC3, BECN1, and ATG5) and apoptotic proteins (CASP9 and BCL2). The Ethics Committee of Public Health Clinical Center Affiliated to Shandong University approved the study (ID: GWLCZXEC-SOP-K-2026 - 15).

### 3.2. Ovariectomy Procedure

To induce estrogen deficiency-associated bone loss, bilateral ovariectomy was performed using a dorsal approach. Anesthesia was induced by intraperitoneal injection of xylazine (5 mg/kg) and ketamine (60 mg/kg) (both from Sigma-Aldrich, St. Louis, MO, USA). Sham-operated animals underwent the same surgical exposure without ovary removal.

### 3.3. Blood Collection and Serum Analyses

At the end of the 10-week treatment period, blood was collected by cardiac puncture, allowed to clot at room temperature, and centrifuged to obtain serum. Serum osteocalcin and estradiol were quantified using a rat-specific sandwich enzyme-linked immunosorbent assay kit (Cloud-Clone Corp., Wuhan, China) according to the manufacturer’s instructions. Serum total calcium was determined using a colorimetric method (Thermo Fisher Scientific, USA), and absorbance was measured at 570 - 575 nm using a microplate reader.

Antioxidant enzyme activities were assessed using standard spectrophotometric assays. Catalase activity was measured by monitoring the decomposition of H_2_O_2_ (Thermo Fisher Scientific, USA) as a decrease in absorbance at 240 nm. *Glutathione reductase* activity was quantified by monitoring nicotinamide adenine dinucleotide phosphate oxidation in the presence of oxidized glutathione (Thermo Fisher Scientific, USA) as a decrease in absorbance at 340 nm, using a UV-visible spectrophotometer.

### 3.4. Histology and Stereological Assessment

Stereological analyses were performed on the entire femur. Before processing, femoral specimens were coded. Image acquisition and quantitative analysis were performed in a blinded manner. After dissection and removal of adherent tissues, femurs allocated to stereology were fixed in 10% formaldehyde (Sigma-Aldrich, St. Louis, MO, USA). Bone volume and microstructural indices were subsequently quantified using unbiased stereological approaches, as described below.

### 3.5. Estimation of Bone Volume

The primary pre-processing femoral volume was estimated using the immersion fluid-displacement method, following a standard Archimedes-based procedure. After determination of the primary volume, femurs were decalcified and processed for routine histology and paraffin embedding. To obtain isotropic, uniformly random sections, femoral sections were generated using the orientator method. For each femur, 8 slabs were prepared. From 1 randomly selected slab, an aspherical/circular fragment was obtained using a trocar, and its area and diameter were recorded before staining. Sections of 5 µm and 20 µm thickness were then prepared from the circular fragment and from all slabs of each femur, with all slabs from a given animal embedded in the same paraffin block.

To correct for processing-related shrinkage, the area of the circular fragment was remeasured after hematoxylin and eosin staining (Sigma-Aldrich, St. Louis, MO, USA), and the degree of shrinkage (Dsh) was calculated as:

$$D_{\mathrm{sh}}=1-({\frac{\mathrm{Are}a_{\mathrm{after}}}{\mathrm{Are}a_{\mathrm{before}})}}^{1.5}$$

The final femoral volume, corrected for shrinkage, was then computed as:

$$V_{\mathrm{final}}=(1-D_{\mathrm{sh}})\times V_{\mathrm{primary}}$$

### 3.6. Trabecular Volume Density and Trabecular Volume

Trabecular bone was quantified using Delesse’s principle and a point-counting method. Briefly, a test-point grid was superimposed on systematically sampled fields, and trabecular volume density (Vv) was estimated as the fraction of test points hitting trabecular bone relative to all test points hitting the reference space. Absolute trabecular volume was then derived by multiplying trabecular volume density by the corrected final femoral volume:

$$V_{\mathrm{trabeculae}}=V_{v_{\mathrm{trabeculae}}}\times V_{\mathrm{final}}$$

### 3.7. Absolute Number of Bone Cells

Numerical density, expressed as cells per unit trabecular volume, and the total number of bone cells were estimated on 20-µm sections using the optical disector approach with an unbiased counting frame. Each 20-µm section was sampled in a systematic-random manner using a microscope equipped with a microcator to control focal depth. Upper and lower guard zones were set at 5 µm each, yielding an effective disector height of 10 µm for counting. Cells were identified morphologically within the trabecular compartment, and nuclei were counted according to unbiased counting rules. A nucleus was counted if it came into focus within the disector height, was fully or partially inside the counting frame, touched the acceptance lines (upper and right borders), and did not touch the rejection lines (lower and left borders). Using optical disector counts and known sampling fractions, numerical density and the total number of cells in the trabecular compartment were calculated using standard stereological formulae.

### 3.8. Femur Processing for Gene Expression and Histology

Femurs were excised, and cartilage and adherent soft tissues were removed. Bone marrow was removed before downstream analyses. For gene expression assays, femoral samples were stored at -80°C until processing. Another femur from each rat was fixed in 10% formaldehyde for histological analyses.

### 3.9. Reverse Transcription Quantitative Polymerase Chain Reaction

RNA extraction and relative expression analyses were conducted using coded samples, and researchers performing molecular analyses remained unaware of group identity until completion of the statistical analysis. RNA extraction was performed using TRIzol (Invitrogen, USA), with protocol optimization for mineralized tissues. RNA quality and quantity were assessed using a spectrophotometer based on the 260/280 absorbance ratio. Complementary DNA was synthesized using a reverse transcription kit (Thermo Fisher Scientific, USA). Real-time quantitative polymerase chain reaction was performed using SYBR Green (Invitrogen, USA) chemistry on an Applied Biosystems 7500 system.

The mRNA expression of autophagy-related targets (LC3, BECN1, and ATG5) and apoptosis-related targets (CASP9 and BCL2) was quantified using gene-specific primers. Relative expression was calculated using the -->FORMULA<-- method and normalized to glyceraldehyde-3-phosphate dehydrogenase. Each reaction was run in technical triplicate. Primer sequences are listed in Table 1.

**Table 1.** Primer Sequences Used for Reverse Transcription Quantitative Polymerase Chain Reaction ^a^

Genes	Forward Primer	Reverse Primer
**LC3**	GAGAAGCAGCTTCCTGTTCTGG	GTGTCCGTTCACCAACAGGAAG
**BECN1**	CTGGACACTCAGCTCAACGTCA	CTCTAGTGCCAGCTCCTTTAGC
**ATG5**	GCAGATGGACAGTTGCACACAC	GAGGTGTTTCCAACATTGGCTCA
**CASP9**	GTTTGAGGACCTTCGACCAGCT	CAACGTACCAGGAGCCACTCTT
**BCL2**	ATCGCCCTGTGGATGACTGAGT	GCCAGGAGAAATCAAACAGAGGC

^a^ Abbreviations: CASP9, caspase 9; BCL2, B-cell lymphoma 2; ATG5, autophagy-related 5; LC3, microtubule-associated protein 1 light chain 3; BECN1, beclin 1.

### 3.10. Statistical Analysis

Data were summarized as mean ± standard error of the mean. Normality and homogeneity of variances were evaluated before between-group comparisons using tests such as the Shapiro-Wilk test for normality and Levene’s or Brown-Forsythe test for equality of variances, and no major violations requiring alternative approaches were observed. Therefore, group differences were assessed using one-way analysis of variance followed by Tukey’s multiple-comparisons test to control the family-wise error rate for pairwise contrasts among SH, OVX, OVX+NA, and OVX+AL. Individual animals served as biological replicates in all statistical tests.

Reverse transcription quantitative polymerase chain reaction was performed in triplicate for each reaction, and the average Ct value for the triplicate reactions was calculated to obtain 1 expression value for each animal before statistical analysis. Other measures, including blood biochemical parameters and stereological, histological, and bone cell assessments, were performed once per animal. All tests were 2-sided, and P < 0.05 was considered statistically significant. Statistical analyses were performed using GraphPad Prism version 9.0 (GraphPad Software, San Diego, CA, USA).

## 4. Results

### 4.1. Stereological and Histomorphometric Outcomes of the Femur

**Figure 1.**
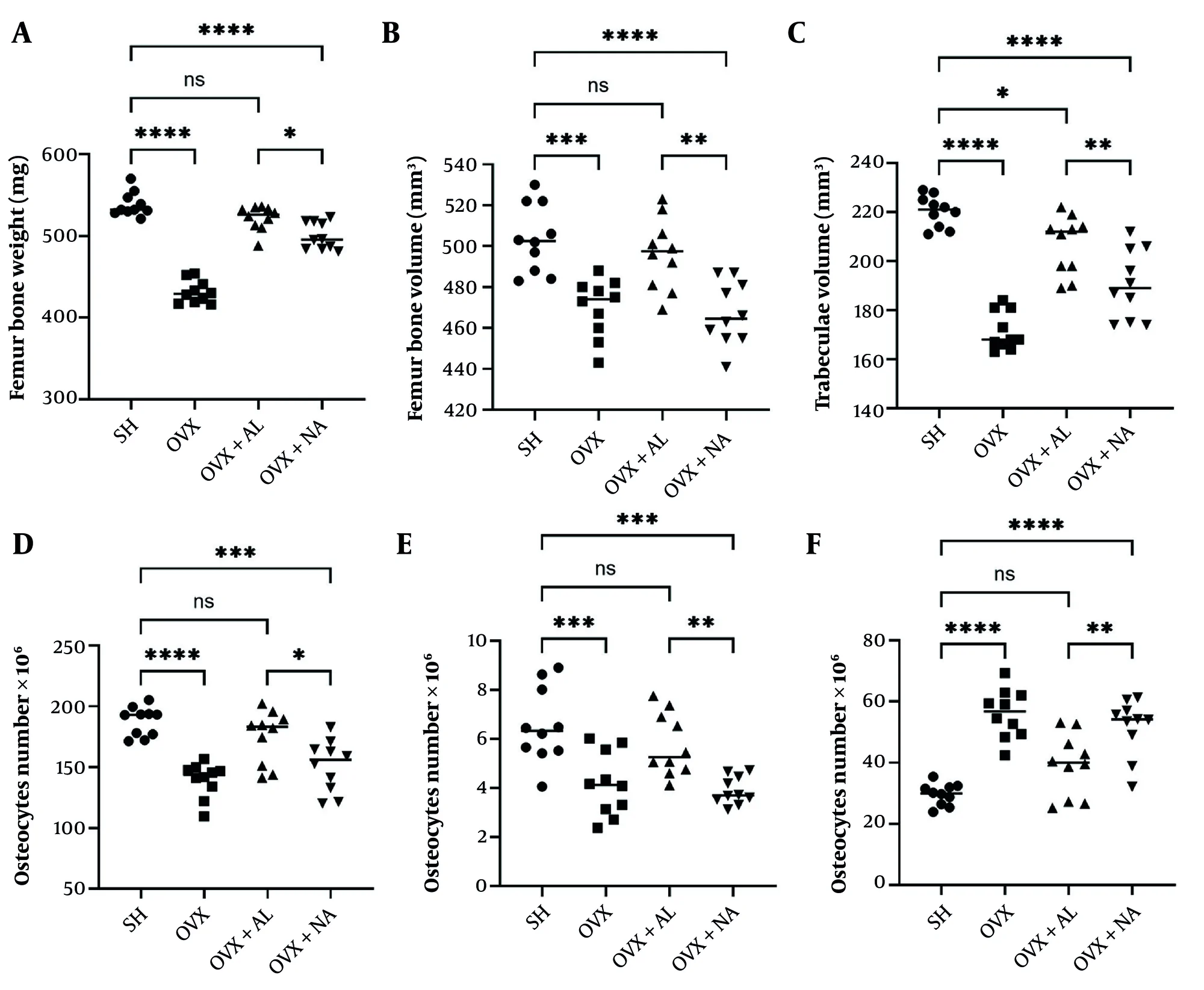
Femoral stereological outcomes in SH, OVX, OVX, OVX+AL, and OVX+NA rats (n = 10/group). (A) Femur bone weight (mg), (B) femur bone volume (mm^3^), (C) trabecular volume (mm^3^), (D) number of osteocytes (scaled as indicated on the y-axis), (E) number of osteoblasts (scaled as indicated on the y-axis), and (F) number of osteoclasts. Data are presented as mean ± standard error of the mean. Statistical analysis: one-way analysis of variance with Tukey’s multiple-comparisons test. *Significance notation*: ns, not significant; *P < 0.05; **P < 0.01; ***P < 0.001; ****P < 0.0001. Abbreviations: AL, alendronate; NA, naringenin; OVX, ovariectomized; SH, sham.

Stereological and histomorphometric analyses of the femur confirmed robust ovariectomy-associated osteoporotic changes. Relative to SH, OVX rats exhibited reduced femur bone weight and total femur bone volume, together with a marked decline in trabecular volume (Figure 1A - C). These structural deficits were accompanied by a coordinated cellular shift within the trabecular compartment, characterized by lower osteocyte and osteoblast numbers and higher osteoclast numbers in OVX rats than in SH rats (Figure 1D - F), consistent with impaired bone maintenance and increased resorptive activity.

**Figure 2.**
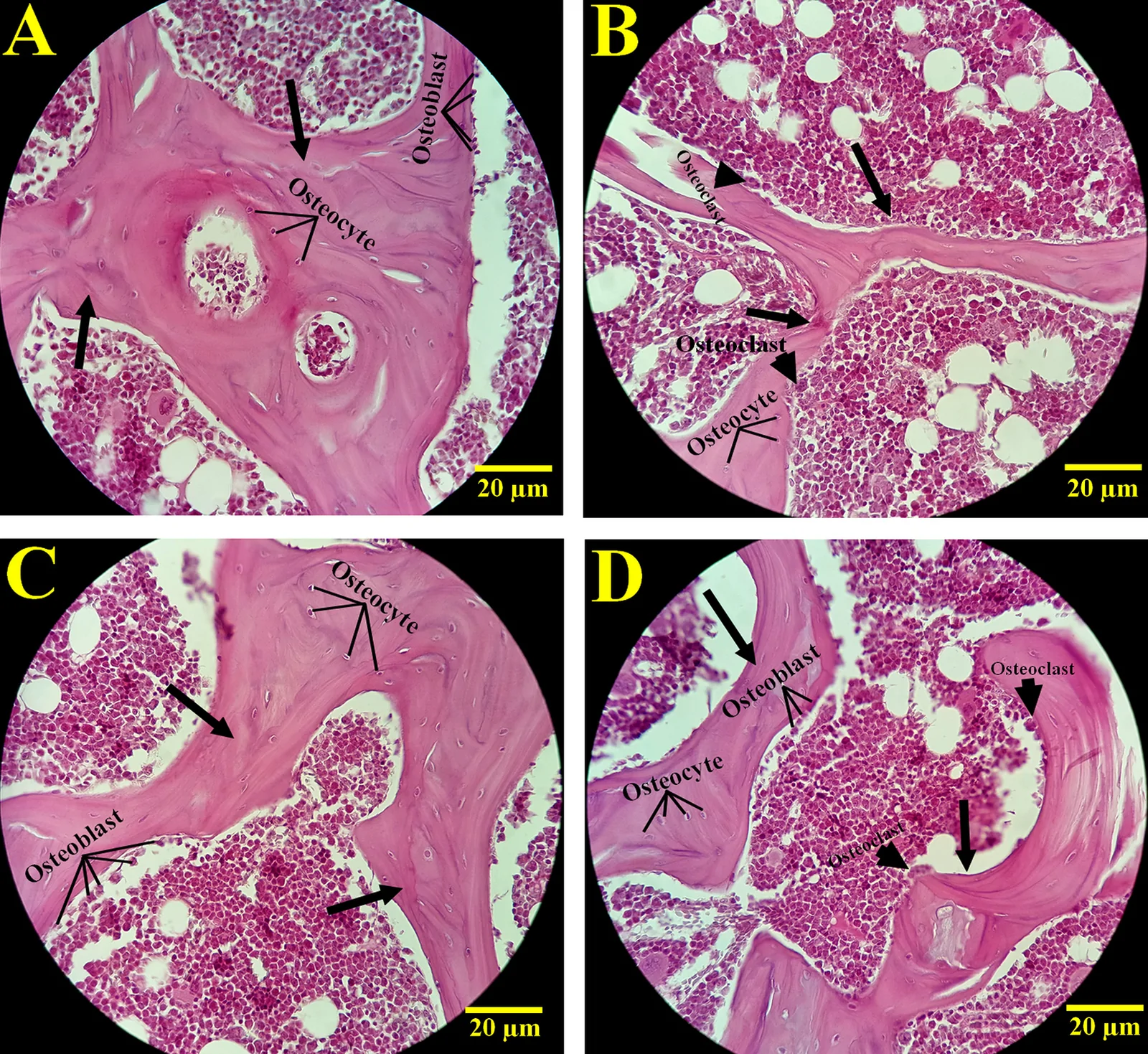
Representative microscopic images of bone sections in all experimental groups. (A) SH, (B) OVX, (C) OVX+AL, and (D) OVX+NA. Sections were stained with hematoxylin and eosin. Abbreviations: AL, alendronate; NA, naringenin; OVX, ovariectomized; SH, sham.

Both interventions attenuated OVX-associated structural and cellular derangements. Overall, OVX+AL and OVX+NA shifted femur bone weight, bone volume, and trabecular volume toward SH values (Figure 1A - C), with parallel normalization of bone cell composition, reflected by increased osteocyte and osteoblast numbers and reduced osteoclast numbers relative to OVX (Figure 1D - F). Across outcomes, improvement was greater in OVX+AL than in OVX+NA, consistent with stronger overall mitigation of OVX-induced osteoporotic changes in the AL-treated group (Figure 1). Representative microscopic images of bone sections from each group are shown in Figure 2.

### 4.2. Serum Biochemical Outcomes

**Figure 3.**
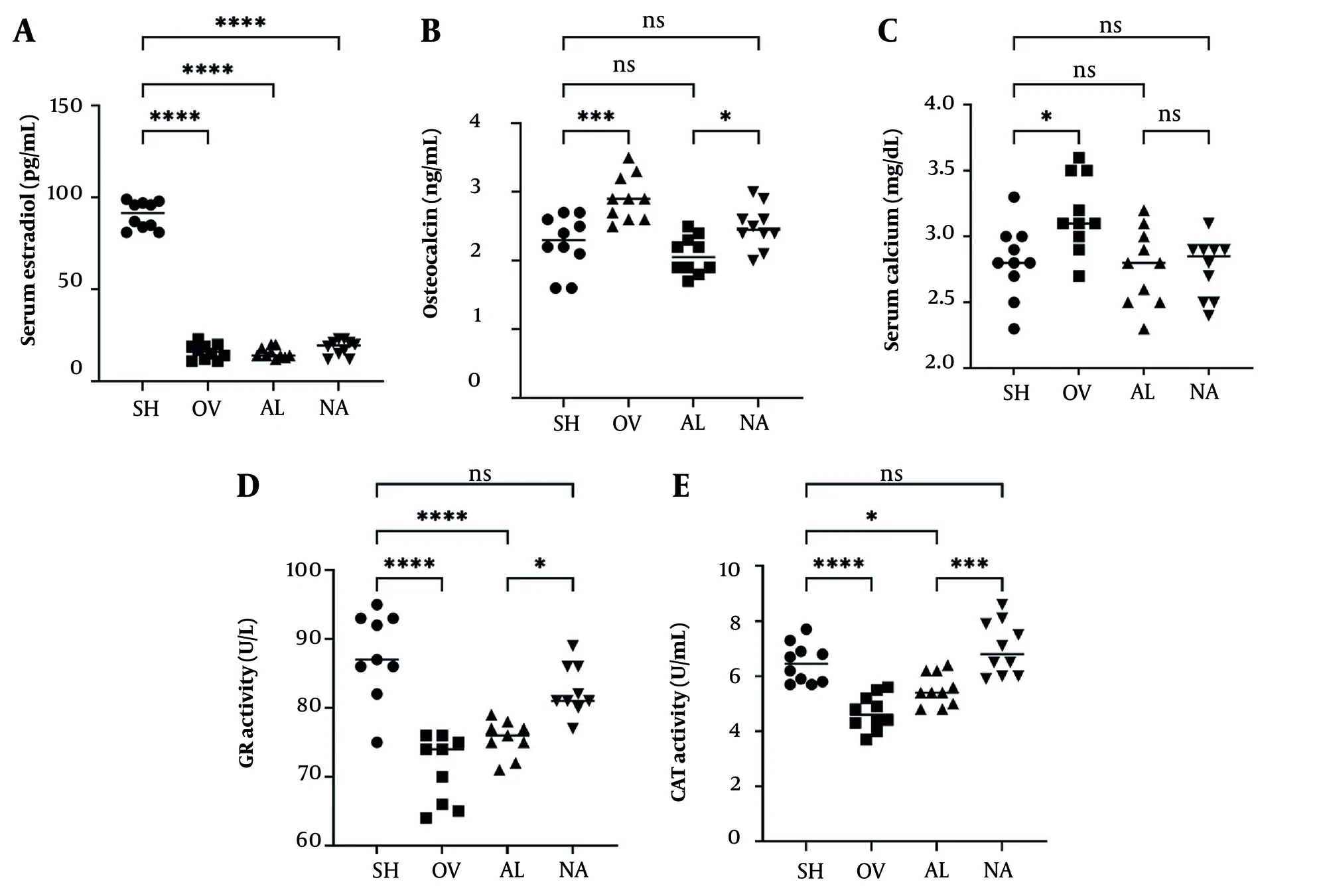
Serum biochemical indices after ovariectomy and treatment in SH, OVX, OVX, OVX+AL, and OVX+NA rats (n = 10/group). (A) Serum estradiol, (B) serum osteocalcin, (C) serum calcium, (D) serum GR activity, and (E) serum CAT activity. Data are shown as individual animals with the group mean (horizontal line); units are as indicated on the y-axes. Statistical analysis: one-way analysis of variance with Tukey’s multiple-comparisons test. *Significance notation*: ns, not significant; *P < 0.05; **P < 0.01; ***P < 0.001; ****P < 0.0001. Abbreviations: AL, alendronate; NA, naringenin; OVX, ovariectomized; SH, sham; GR, glutathione reductase; CAT, catalase.

As expected, ovariectomy was associated with a marked reduction in circulating estradiol relative to SH (mean difference SH - OVX = 74.3; Tukey-adjusted P < 0.0001). Importantly, estradiol remained comparably low in OVX+AL and OVX+NA compared with OVX (OVX vs AL: P = 0.9929; OVX vs NA: P = 0.7532), indicating that neither intervention restored systemic estradiol within the treatment window (Figure 3A).

Serum osteocalcin differed significantly between SH and OVX (mean difference SH - OVX = -0.65; P = 0.0006). Both interventions shifted osteocalcin relative to OVX, with lower values in OVX+AL (OVX vs AL: P < 0.0001) and OVX+NA (OVX vs NA: P = 0.0380). Osteocalcin was also lower in OVX+AL than in OVX+NA (AL vs NA: P = 0.0445), consistent with more pronounced modulation in the AL-treated group (Figure 3B).

Serum calcium differed between SH and OVX (mean difference SH - OVX = -0.36; P = 0.0263). Calcium values in OVX+AL and OVX+NA did not differ from SH (SH vs AL: P = 0.9875; SH vs NA: P = 0.9761) and were lower than those in OVX (OVX vs AL: P = 0.0114; OVX vs NA: P = 0.0092), suggesting normalization of calcium homeostasis by both treatments under the conditions tested (Figure 3C).

Antioxidant enzyme indices indicated an ovariectomy-associated decrement in redox defense. *Glutathione reductase* was lower in OVX than in SH (mean difference SH - OVX = 16.56; P < 0.0001). OVX+NA showed higher glutathione reductase than OVX (OVX vs NA: P < 0.0001), whereas OVX+AL did not differ from OVX (OVX vs AL: P = 0.1979). *Glutathione reductase* remained lower in OVX+AL than in SH (SH vs AL: P < 0.0001), and OVX+AL was lower than OVX+NA (AL vs NA: P = 0.0156) (Figure 3D).

A similar pattern was observed for CAT. Catalase was lower in OVX than in SH (mean difference SH - OVX = 1.79; P < 0.0001) and higher in OVX+NA than in OVX (OVX vs NA: P < 0.0001). In contrast, the difference between OVX and OVX+AL did not meet the adjusted significance threshold (OVX vs AL: P = 0.0713), and CAT was lower in OVX+AL than in SH (SH vs AL: P = 0.0333), as well as lower in OVX+AL than in OVX+NA (AL vs NA: P = 0.0004) (Figure 3E). Collectively, these data support a clearer recovery of antioxidant enzyme activity in the NA-treated group under this experimental design.

### 4.3. Bone Gene Expression: Apoptosis- and Autophagy-Related Markers

**Figure 4.**
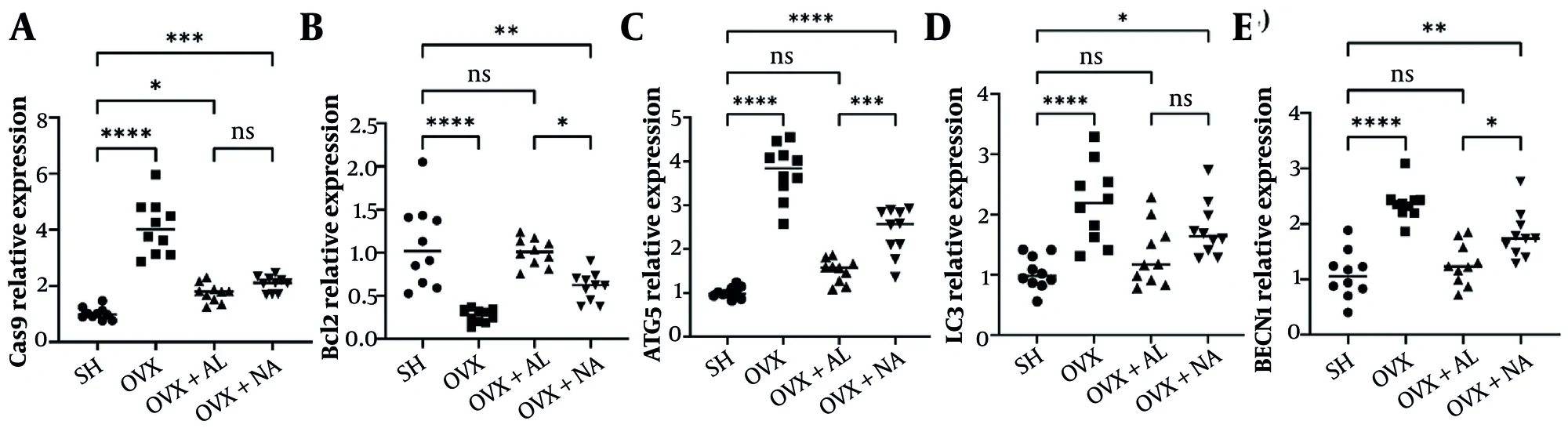
Femoral apoptosis- and autophagy-related gene expression, assessed by reverse transcription quantitative polymerase chain reaction, in SH, OVX, OVX+AL, and OVX+NA rats (n = 10/group). (A) CASP9, (B) BCL2, (C) ATG5, (D) LC3, and (E) BECN1. Data are shown as individual animals with the group mean (horizontal line). Statistical analysis: one-way analysis of variance with Tukey’s multiple-comparisons test. *Significance notation*: ns, not significant; *P < 0.05; **P < 0.01; ***P < 0.001; ****P < 0.0001. Abbreviations: AL, alendronate; NA, naringenin; OVX, ovariectomized; SH, sham; CASP9, caspase 9; BCL2, B-cell lymphoma 2; ATG5, autophagy-related 5; LC3, microtubule-associated protein 1 light chain 3; BECN1, beclin 1.

Ovariectomy was associated with a pro-apoptotic shift in the assessed transcript markers. CASP9 expression was higher in OVX than in SH (mean difference SH - OVX = -3.062; P < 0.0001). Both treatments reduced CASP9 relative to OVX (OVX vs AL: P < 0.0001; OVX vs NA: P < 0.0001), although CASP9 remained higher than SH in both OVX+AL (SH vs AL: P = 0.0222) and OVX+NA (SH vs NA: P = 0.0006). CASP9 did not differ between OVX+AL and OVX+NA (AL vs NA: P = 0.5497) (Figure 4A).

Conversely, BCL2 expression was lower in OVX than in SH (mean difference SH - OVX = 0.8220; P < 0.0001). Both interventions increased BCL2 relative to OVX (OVX vs AL: P < 0.0001; OVX vs NA: P = 0.0396). BCL2 in OVX+AL did not differ from SH (SH vs AL: P = 0.8901), whereas BCL2 in OVX+NA remained lower than SH (SH vs NA: P = 0.0015). BCL2 was higher in OVX+AL than in OVX+NA (AL vs NA: P = 0.0106), suggesting more complete restoration toward SH levels with AL for this marker (Figure 4B).

Autophagy-related transcripts were also altered by OVX. ATG5, LC3, and BECN1 each differed between SH and OVX (ATG5: mean difference SH - OVX = -2.752, P < 0.0001; LC3: SH - OVX = -1.146, P < 0.0001; BECN1: SH - OVX = -1.291, P < 0.0001). For ATG5, both treatments reduced expression relative to OVX (OVX vs AL: P < 0.0001; OVX vs NA: P < 0.0001), with lower ATG5 in OVX+AL than in OVX+NA (AL vs NA: P = 0.0003). ATG5 in OVX+AL approached SH without meeting the adjusted threshold (SH vs AL: P = 0.0700), whereas ATG5 remained higher than SH in OVX+NA (SH vs NA: P < 0.0001) (Figure 4C).

For LC3, OVX+AL was lower than OVX (OVX vs AL: P = 0.0025) and did not differ from SH (SH vs AL: P = 0.5585). LC3 in OVX+NA was higher than SH (SH vs NA: P = 0.0138) and did not differ significantly from OVX (OVX vs NA: P = 0.2246), indicating a more modest or variable shift with NA for this transcript in the present dataset (Figure 4D). For BECN1, both OVX+AL and OVX+NA were lower than OVX (OVX vs AL: P < 0.0001; OVX vs NA: P = 0.0109), yet BECN1 remained higher than SH in both treatment groups (SH vs AL: P = 0.6988; SH vs NA: P = 0.0011), with BECN1 lower in OVX+AL than in OVX+NA (AL vs NA: P = 0.0215) (Figure 4E).

Overall, the reverse transcription quantitative polymerase chain reaction profile indicates that OVX was accompanied by coordinated dysregulation of apoptosis- and autophagy-linked transcripts and that AL generally produced greater normalization toward SH patterns than NA across the assessed autophagy markers and BCL2. However, these findings reflect transcriptional associations and should not be interpreted as direct evidence of functional pathway modulation without protein-level and flux validation.

## 5. Discussion

In this OVX model of estrogen deficiency-associated bone loss, 10 weeks of oral NA or AL attenuated the biochemical, molecular, and structural hallmarks of osteoporotic change. Across evaluated endpoints, AL showed more consistent normalization across multiple structural and molecular outcomes, whereas NA showed a stronger association with the restoration of antioxidant enzyme activities. Importantly, neither NA nor AL restored circulating estradiol relative to OVX, supporting a mechanism that is largely independent of systemic estrogen replacement and more consistent with downstream modulation of bone remodeling and cellular stress-response pathways than with systemic estrogen replacement (4, 6, 18).

First, OVX increased the high-turnover profile reflected by serum osteocalcin, and both interventions reduced this elevation, with AL showing a greater corrective effect than NA (OVX vs AL: adjusted P < 0.0001; OVX vs NA: adjusted P = 0.0380; AL vs NA: adjusted P = 0.0445). This pattern aligns with established antiresorptive pharmacodynamics, in which suppression of osteoclast activity reduces coupled remodeling and lowers circulating turnover markers, including osteocalcin, even when bone formation at the tissue level ultimately improves through preservation of trabecular structure (34, 35). Serum calcium differences followed a similar direction: OVX differed from SH (adjusted P = 0.0263), and both AL and NA shifted calcium toward SH levels (OVX vs AL: adjusted P = 0.0114; OVX vs NA: adjusted P = 0.0092), consistent with partial correction of remodeling-driven calcium efflux under estrogen deficiency.

Second, the stereological/histomorphometric dataset (Figure 1) indicates that OVX compromised bone mass and trabecular architecture and altered bone cell numbers, whereas both treatments mitigated these structural and cellular deficits. Although stereological outputs are often reported as volumetric and numerical-density-derived indices rather than areal proxies, their biological interpretation aligns with the canonical OVX phenotype: trabecular rarefaction, reduced osteoblast and osteocyte representation, and increased osteoclast presence as remodeling becomes imbalanced (30). Within this framework, the stronger global improvement observed with AL is biologically plausible given the established efficacy of nitrogen-containing bisphosphonates in suppressing osteoclast-mediated resorption through inhibition of farnesyl pyrophosphate synthase in the mevalonate pathway, which disrupts prenylation-dependent osteoclast function and promotes osteoclast apoptosis (36). Therefore, the structural recovery in the AL group is consistent with a primary antiresorptive effect that secondarily stabilizes trabecular microarchitecture.

Third, NA exhibited a distinctive signature in systemic antioxidant defenses. Ovariectomy markedly reduced CAT and GR activities compared with SH (both adjusted P < 0.0001), consistent with oxidative stress as a contributor to post-OVX bone deterioration (37, 38). Naringenin significantly increased both enzymes relative to OVX (GR: adjusted P < 0.0001; CAT: adjusted P < 0.0001), and values in the NA group did not differ from SH for either GR (adjusted P = 0.1110) or CAT (adjusted P = 0.3753). In contrast, AL improved neither GR (OVX vs AL: adjusted P = 0.1979) nor CAT (OVX vs AL: adjusted P = 0.0713) to the same extent, and both GR and CAT remained significantly different from SH in the AL group (adjusted P < 0.0001 and P = 0.0333, respectively). The present findings therefore support a model in which the benefits of NA may be partly mediated by restoration of systemic redox capacity, complementing its direct effects on bone cell biology described in the experimental osteoporosis literature (24, 28).

At the molecular level, OVX induced a coordinated shift in apoptosis- and autophagy-related transcription within femoral tissue (Figure 4). Ovariectomy increased CASP9 expression relative to SH (SH vs OVX mean difference = -3.062; adjusted P < 0.0001) and reduced BCL2 expression (SH vs OVX mean difference = 0.8220; adjusted P < 0.0001), implicating engagement of mitochondrial apoptosis signaling under estrogen deficiency. Osteocyte and osteoblast apoptosis are increasingly recognized as mechanistic amplifiers of bone loss, as osteocyte death can promote targeted remodeling and propagate pro-resorptive cues within the bone microenvironment (39). Against this backdrop, both interventions shifted apoptosis markers toward SH values, with AL and NA reducing CASP9 relative to OVX (both adjusted P < 0.0001) and increasing BCL2 relative to OVX (AL: adjusted P < 0.0001; NA: adjusted P = 0.0396). Notably, BCL2 differed between AL and NA (adjusted P = 0.0106), suggesting greater normalization by AL at the transcriptional level in this dataset.

Autophagy-related transcripts, including ATG5, LC3B, and BECN1, were also elevated in OVX compared with SH (all adjusted P < 0.0001), indicating that estrogen deficiency in this model was accompanied by activation or remodeling of autophagy-associated gene programs. Autophagy is tightly integrated into bone cell survival and differentiation, and its dysregulation, whether insufficient protective autophagy or maladaptive/excessive activation, can disturb the balance between osteogenesis and resorption (12-14). Importantly, increased osteocyte autophagy has been reported in OVX rodents and has been linked to oxidative stress-related alterations in the femur, supporting the plausibility that the observed transcriptional elevation represents a stress-adaptive response rather than a straightforward beneficial increase in autophagic flux (40). In the current study, AL reduced ATG5 and BECN1 to a greater degree than NA (AL vs NA: ATG5, adjusted P = 0.0003; BECN1, adjusted P = 0.0215) and restored these markers closer to SH values, with SH vs AL not significant for ATG5 and BECN1. In contrast, NA remained significantly different from SH for ATG5, LC3B, and BECN1.

A key unresolved point is whether transcriptional changes correspond to functional autophagic flux in bone cells. Without protein-level markers, such as LC3-II/LC3-I ratios, and flux assays, increased expression could also reflect impaired completion of autophagy with compensatory upregulation (12, 13). Integrating the biochemical and transcriptional layers suggests a coherent mechanistic axis: OVX-associated oxidative stress, reflected by CAT and GR suppression, coincides with increased autophagy-related transcription and a shift toward mitochondrial apoptosis, indicated by increased CASP9 and decreased BCL2. Together, these changes can impair osteoblast and osteocyte viability and favor osteoclast-driven resorption (13, 37-39). These alterations likely converge at the tissue level to drive stereological/histomorphometric outcomes reflecting trabecular structure and bone cell balance.

Several strengths support the interpretability of these findings. The study combined circulating biochemical indices, femoral stereology/histomorphometry, and targeted reverse transcription quantitative polymerase chain reaction, allowing cross-validation of remodeling, structural, and pathway-level signals. The use of unbiased stereological principles, including orientator/disector-based quantification, is particularly valuable for estimating cell numbers, reducing geometric and sampling bias relative to purely areal histology readouts.

Nonetheless, limitations constrain mechanistic certainty. Only transcript-level endpoints were measured for autophagy and apoptosis, and functional confirmation at the protein or flux level was not performed. Therefore, our observations should be interpreted as suggesting a correlation with aberrant signaling through these pathways rather than proof of modulation of autophagy or apoptosis (12-14). The lack of protein-based evidence, such as LC3-II/LC3-I ratios, beclin-1 protein levels, cleaved caspases, or autophagic flux studies, leaves unanswered whether the transcriptional changes indicate beneficial upregulation, detrimental buildup, or adaptive responses to another stressor. Within the parameters of the present experimental setting, AL showed stronger beneficial effects than NA. However, this assessment of AL should not be generalized to its overall evaluation, and no conclusion about the ultimate superiority of AL over NA can be made. Dynamic histomorphometry, such as fluorochrome labeling, micro-computed tomography, and biomechanical testing, was not included, and these methods would strengthen claims regarding bone strength and microarchitecture. Finally, combined NA+AL therapy was not evaluated; therefore, any inference about additive or synergistic benefit remains speculative and should be reserved for future factorial studies.

From a translational perspective, AL remains the more potent intervention across integrated outcomes, consistent with its established clinical efficacy as a first-line antiresorptive agent (34-36). The profile of NA, particularly its normalization of antioxidant enzymes alongside partial correction of apoptosis/autophagy transcription, supports further evaluation as an adjunctive strategy aimed at mitigating oxidative stress-linked bone cell vulnerability in estrogen-deficiency states (24, 28, 37, 38).

Future work should prioritize 1) validation of autophagic flux and apoptosis-related proteins in bone compartments, 2) longitudinal microstructural and mechanical endpoints, and 3) combination or sequential regimens to test whether NA can complement bisphosphonate therapy without compromising remodeling suppression.

### 5.1. Conclusions

In an ovariectomy-induced model of estrogen deficiency-associated bone loss, 10 weeks of oral NA or AL mitigated osteoporotic changes across circulating biochemical indices, femoral gene expression signatures linked to apoptosis and autophagy, and stereological/histomorphometric outcomes. Neither intervention restored serum estradiol, supporting an effect that is largely independent of systemic estrogen recovery. These results suggest that the improving effects of AL and NA may be associated with modulation of oxidative stress, apoptosis, and autophagy-related mechanisms.

### 5.2. Clinical/Translational Implications

These findings support that AL may exert anti-osteoporotic effects in this experimental setting and also suggest that NA may provide complementary benefits by improving redox-related defenses and partially correcting stress-response pathways in bone. Naringenin therefore warrants further study as a potential adjunct strategy, particularly in designs that incorporate functional bone outcomes, such as micro-computed tomography and biomechanical testing, and mechanistic confirmation of autophagic flux and apoptotic signaling at the protein level.

## Data Availability

The data that support the findings of this study are available from the corresponding author upon reasonable request.

## References

[1] Chin KY, Ng BN, Rostam MKI, Muhammad Fadzil NFD, Raman V, Mohamed Yunus F (2022). A mini review on osteoporosis: from biology to pharmacological management of bone loss. Journal of Clinical Medicine.

[2] Zhao H, Yu F, Wu W (2025). New perspectives on postmenopausal osteoporosis: mechanisms and potential therapeutic strategies of sirtuins and oxidative stress. Antioxidants.

[3] Lee KIR, Chen JH, Chen KH (2026). Osteoporosis after menopause and after drug therapy: the molecular mechanism of bone loss and its treatment. International Journal of Molecular Sciences.

[4] Gregson CL, Armstrong DJ, Bowden J, Cooper C, Edwards J, Gittoes NJL (2022). UK clinical guideline for the prevention and treatment of osteoporosis. Archives of Osteoporosis.

[5] Qaseem A, Owens DK, Etxeandia-Ikobaltzeta I, Tufte J, Cross JT, Wilt TJ (2023). Pharmacologic treatment of primary osteoporosis or low bone mass to prevent fractures in adults: a living clinical guideline from the American College of Physicians. Annals of Internal Medicine.

[6] Morin SN, Feldman S, Funnell L, Giangregorio L, Kim S, McDonald-Blumer H (2023). Clinical practice guideline for management of osteoporosis and fracture prevention in Canada: 2023 update. CMAJ.

[7] Li L, Sun Y, Luo J, Liu M (2024). Circulating immune cells and risk of osteosarcoma: a Mendelian randomization analysis. Frontiers in Immunology.

[8] Hsu SH, Chen LR, Chen KH (2024). Primary osteoporosis induced by androgen and estrogen deficiency: the molecular and cellular perspective on pathophysiological mechanisms and treatments. International Journal of Molecular Sciences.

[9] Luo J, Li L, Shi W, Xu K, Shen Y, Dai B (2025). Oxidative stress and inflammation: roles in osteoporosis. Frontiers in Immunology.

[10] Li WQ, Wu JY, Xiang DX, Luo SL, Hu XB, Tang TT (2019). Micelles loaded with puerarin and modified with triphenylphosphonium cation possess mitochondrial targeting and demonstrate enhanced protective effect against isoprenaline-induced H9c2 cells apoptosis. International Journal of Nanomedicine.

[11] Amroodi MN, Maghsoudloo M, Amiri S, Mokhtari K, Mohseni P, Pourmarjani A (2024). Unraveling the molecular and immunological landscape: exploring signaling pathways in osteoporosis. Biomedicine & Pharmacotherapy.

[12] Yin X, Zhou C, Li J, Liu R, Shi B, Yuan Q (2019). Autophagy in bone homeostasis and the onset of osteoporosis. Bone Research.

[13] Zhu C, Shen S, Zhang S, Huang M, Zhang L, Chen X (2022). Autophagy in bone remodeling: a regulator of oxidative stress. Frontiers in Endocrinology.

[14] Li X, Xu J, Dai B, Wang X, Guo Q, Qin L (2020). Targeting autophagy in osteoporosis: from pathophysiology to potential therapy. Ageing Research Reviews.

[15] Ebrahimi K, Soori R, Badalzadeh R, Baghaiee B, Hemayattalab A (2025). High-intensity interval training protectively modulates doxorubicin-induced changes in cardiac mitochondrial dynamics and autophagy process. Journal of Clinical Research in Paramedical Sciences.

[16] Wang W, Zhang LM, Guo C, Han JF (2020). Resveratrol promotes osteoblastic differentiation in a rat model of postmenopausal osteoporosis by regulating autophagy. Nutrition & Metabolism.

[17] Wei L, Chai S, Yue C, Zhang H, Li J, Qin N (2023). Resveratrol protects osteocytes against oxidative stress in ovariectomized rats through AMPK/JNK1-dependent pathway leading to promotion of autophagy and inhibition of apoptosis. Cell Death Discovery.

[18] Papapoulos SE, Makras P (2025). Bisphosphonates in the management of patients with postmenopausal osteoporosis; back to the future. Pharmaceuticals.

[19] Kuroshima S, Sasaki M, Sawase T (2019). Medication-related osteonecrosis of the jaw: a literature review. Journal of Oral Biosciences.

[20] El-Rabbany M, Sgro A, Lam DK, Shah PS, Azarpazhooh A (2017). Effectiveness of treatments for medication-related osteonecrosis of the jaw: a systematic review and meta-analysis. The Journal of the American Dental Association.

[21] Perrone P, De Rosa C, D’Angelo S (2025). Polyphenols and bone health: a comprehensive review of their role in osteoporosis prevention and treatment. Molecules.

[22] Ghanbari A, Jalili C, Shahveisi K, Raissi F, Mazini F, Akhshi N (2023). Antioxidant, anti-apoptotic and anti-inflammatory effects of acacetin against deltamethrin-induced hepatotoxicity in mice: a biochemical and histopathological study. Koomesh.

[23] Dorostghoal M, Dayer M, Biabi N (2025). Protective effects of taurine against 5-fluorouracil induced testicular oxidative toxicity in male Wistar rats. Gene, Cell and Tissue.

[24] Zhou X, Zhang Z, Jiang W, Hu M, Meng Y, Li W (2022). Naringenin is a potential anabolic treatment for bone loss by modulating osteogenesis, osteoclastogenesis, and macrophage polarization. Frontiers in Pharmacology.

[25] Nor Muhamad ML, Ekeuku SO, Wong SK, Chin KY (2022). A scoping review of the skeletal effects of naringenin. Nutrients.

[26] Javadiyeh A, Heidari Kaydan H, Bagheri F, Goodarzi S, Salimi A (2026). Design, preparation, and ex vivo skin permeation of naringenin liposomal formulation for topical delivery. Jundishapur Journal of Natural Pharmaceutical Products.

[27] Zhou J, Li H, Wu B, Zhu L, Huang Q, Guo Z (2024). Network pharmacology combined with experimental verification to explore the potential mechanism of naringenin in the treatment of cervical cancer. Scientific Reports.

[28] Kaczmarczyk-Sedlak I, Wojnar W, Zych M, Ozimina-Kamińska E, Bońka A (2016). Effect of dietary flavonoid naringenin on bones in rats with ovariectomy-induced osteoporosis. Acta Poloniae Pharmaceutica.

[29] Zhu Z, Xie W, Li Y, Zhu Z, Zhang W (2021). Effect of naringin treatment on postmenopausal osteoporosis in ovariectomized rats: a meta-analysis and systematic review. Evidence-Based Complementary and Alternative Medicine.

[30] Yousefzadeh N, Kashfi K, Jeddi S, Ghasemi A (2020). Ovariectomized rat model of osteoporosis: a practical guide. EXCLI Journal.

[31] Gorji-Valokola M, Babaloo H, Kheradmand H (2022). Effect of human adipose-derived mesenchymal stem cells preconditioned with cobalt chloride for hypoxia induction on ovariectomy-induced osteoporosis. Koomesh.

[32] Xu Z, Huang B, Liu J, Wu X, Luo N, Wang X (2018). Combinatorial anti-proliferative effects of tamoxifen and naringenin: the role of four estrogen receptor subtypes. Toxicology.

[33] Montazeri-Najafabady N, Ghasemi Y, Dabbaghmanesh MH, Talezadeh P, Koohpeyma F, Gholami A (2019). Supportive role of probiotic strains in protecting rats from ovariectomy-induced cortical bone loss. Probiotics and Antimicrobial Proteins.

[34] Schini M, Vilaca T, Gossiel F, Salam S, Eastell R (2023). Bone turnover markers: basic biology to clinical applications. Endocrine Reviews.

[35] Brown JP, Don-Wauchope A, Douville P, Albert C, Vasikaran SD (2022). Current use of bone turnover markers in the management of osteoporosis. Clinical Biochemistry.

[36] Scala R, Maqoud F, Antonacci M, Dibenedetto JR, Perrone MG, Scilimati A (2022). Bisphosphonates targeting ion channels and musculoskeletal effects. Frontiers in Pharmacology.

[37] Zhang C, Li H, Li J, Hu J, Yang K, Tao L (2023). Oxidative stress: a common pathological state in a high-risk population for osteoporosis. Biomedicine & Pharmacotherapy.

[38] Marcucci G, Domazetovic V, Nediani C, Ruzzolini J, Favre C, Brandi ML (2023). Oxidative stress and natural antioxidants in osteoporosis: novel preventive and therapeutic approaches. Antioxidants.

[39] Ru JY, Wang YF (2020). Osteocyte apoptosis: the roles and key molecular mechanisms in resorption-related bone diseases. Cell Death Dis.

[40] Xiong Y, Huang CW, Shi C, Peng L, Cheng YT, Hong W (2023). Quercetin suppresses ovariectomy-induced osteoporosis in rat mandibles by regulating autophagy and the NLRP3 pathway. Experimental Biology and Medicine.

